# Comparison of *in vitro* static and dynamic assays to evaluate the efficacy of an antimicrobial drug combination against *Staphylococcus aureus*

**DOI:** 10.1371/journal.pone.0211214

**Published:** 2019-01-23

**Authors:** Diane C. Broussou, Pierre-Louis Toutain, Frédérique Woehrlé, Farid El Garch, Alain Bousquet-Melou, Aude A. Ferran

**Affiliations:** 1 UMR 1436 INTHERES, Université de Toulouse, INRA, ENVT, Toulouse, France; 2 Vetoquinol SA, Lure, France; 3 Department of Veterinary Basics Sciences, Royal Veterinary College, London, United Kingdom; Universitatsklinikum Munster, GERMANY

## Abstract

An easily implementable strategy to reduce treatment failures in severe bacterial infections is to combine already available antibiotics. However, most *in vitro* combination assays are performed by exposing standard bacterial inocula to constant concentrations of antibiotics over less than 24h, which can be poorly representative of clinical situations. The aim of this study was to assess the ability of static and dynamic *in vitro* Time-Kill Studies (TKS) to identify the potential benefits of an antibiotic combination (here, amikacin and vancomycin) on two different inoculum sizes of two *S*. *aureus* strains. In the static TKS (sTKS), performed by exposing both strains over 24h to constant antibiotic concentrations, the activity of the two drugs combined was not significantly different the better drug used alone. However, the dynamic TKS (dTKS) performed over 5 days by exposing one strain to fluctuating concentrations representative of those observed in patients showed that, with the large inoculum, the activities of the drugs, used alone or in combination, significantly differed over time. Vancomycin did not kill bacteria, amikacin led to bacterial regrowth whereas the combination progressively decreased the bacterial load. Thus, dTKS revealed an enhanced effect of the combination on a large inoculum not observed in sTKS. The discrepancy between the sTKS and dTKS results highlights that the assessment of the efficacy of a combination for severe infections associated with a high bacterial load could be demanding. These situations probably require the implementation of dynamic assays over the entire expected treatment duration rather than the sole static assays performed with steady drug concentrations over 24h.

## Introduction

The recalcitrance of severe, chronic bacterial infections to antibiotic treatments, due to tolerance or resistance, is necessitating a search for new antimicrobial strategies. One therapeutic option to extend the spectrum, minimize the emergence of resistance or enhance the bactericidal activity, and which can be easily and rapidly implemented, is to use a combination of existing antimicrobials [1,2]. However, the potential advantage of the combination for managing an infection has to be assessed *in vitro* before its implementation in clinical practice. Currently, one of the most commonly used *in vitro* assays to evaluate the efficacy of a combination is the checkerboard assay (CA). This is based on a similar method to the one used for MIC determination and involves exposure of a standard inoculum of 5.7 log_10_ CFU/mL [3] to constant concentrations of one or two drugs followed by assessment of the turbidity after incubating the mixture for 16–20 hours. These CA results are used to classify the combination in one of three categories: synergy, indifference or antagonism [4]. Another more time-consuming method is to carry out static time-kill studies (sTKS) in which constant concentrations of drugs are used to monitor the kinetics of bacterial killing over time [5–8]. sTKS is a more quantitative approach than CA as the bacterial counts during exposure to the drugs alone or in combination are compared over 24 h. However, although both CA and sTKS assays are inexpensive and quite easy to perform, their limitations include short duration of the assay and the exposure to constant concentrations of drugs. Indeed, due to the absence of broth renewal, both assays take less than 24 hours, which can prevent the detection of potential bacterial regrowth, predictive of a relapse in the patient [9], or of progressive activity leading to bacterial eradication after several days of treatment. Thus, an exposure to constant concentrations does not reflect the behaviour of drugs in patients since the phenomena of drug absorption, metabolism and elimination lead to fluctuating concentrations over time and to a potential exposure of bacteria to sub-inhibitory concentrations which can promote the development of resistance [10]. A Hollow-Fiber (HF) infection model has been proposed to overcome these limitations [11]. This model is used in dynamic time-kill studies (dTKS) to follow the evolution of a bacterial population exposed to antibiotic concentrations that are similar to those observed in patients treated over several days.

Although many studies of antibiotic combinations have focused on standard inoculum sizes, infections that are refractory to treatments and require an improved antibiotic strategy are mainly associated with biofilms and a large bacterial load. Moreover, an inoculum effect that reduces antibacterial activity on large bacterial population has been described for many drugs including vancomycin against inoculum sizes of *Staphylococcus (S*.*) aureus* strains ranging from 8 to 9.5 log_10_ CFU/mL [12,13]. We can therefore conclude that *in vitro* assessments of antibiotic combinations need to be performed in conditions that could more accurately predict their efficacy against severe refractory infections associated with large inocula. Besides, the use of Mueller-Hinton Broth (MHB) was shown to be poorly predictive of *in vivo* outcomes for some specific cases [14].

The aim of this study was not to promote the use of one specific combination but rather to detect discrepancies in the assessments of the efficacy of the same antibiotic combination by using different methods, culture media or inoculum sizes. Thus, we conducted the experiments with one selected combination to test our hypothesis which was the combination of vancomycin and an aminoglycoside recommended as the empirical treatment of some natural and prosthetic cardiac endocarditis [15]. The combination of vancomycin and amikacin was also previously investigated on *S*. *aureus* under static conditions [16].

We compared the efficacy of this combination on standard (around 5 log_10_ CFU/mL) and large (around 9 log_10_ CFU/mL) inocula of two *S*. *aureus* strains by performing CA, sTKS and dTKS in different media. For sTKS, the inocula were exposed for 24 hours to the maximum free plasma concentrations obtained in patients at the current dosing regimen, i.e. after one administration of 15mg/kg amikacin [17] and/or 1 g of vancomycin by IV route [18]. For dTKS in a HF model, the inocula were exposed for 5 days to fluctuating concentrations, as could be observed in patients treated once a day with amikacin and/or every 12h with vancomycin [19,20].

## Material and methods

### Bacterial strains

A Methicillin Resistant *S*. *aureus* (MRSA) ATCC 33591 and a Methicillin Susceptible *S*. *aureus* MSSA (MSSA) HG001, derived from NCTC 8325, were used for the CA and for sTKS with constant antibiotic concentrations. The MSSA strain HG001 was used for the dTKS with fluctuating antibiotic concentrations in the HF model. Stock cultures of the bacteria were preserved at -80°C in MHB (Sigma-Aldrich, Saint Quentin-Fallavier, France) supplemented with 15% glycerol. Before each assay, 10μL of the bacterial suspension was incubated overnight at 37°C on a Mueller-Hinton agar plate.

### Antimicrobial agents

Amikacin sulfate powder (Amikacine Mylan) and vancomycin chlorhydrate powder (Vancomycin Sandoz) for intravenous administration were used to prepare antibiotic stock solutions in water at desired concentrations and stored at -20°C for less than 1 month.

Antibiotic solutions were thawed and diluted to desired concentrations just before use.

### Minimal inhibitory concentration (MIC) determination

The MICs of vancomycin and amikacin for the MRSA and MSSA strains were performed in three independent experiments by broth microdilution with cation adjusted-MHB (Mueller-Hinton II, Sigma Aldrich, Saint-Quentin-Fallavier, France) according to the CLSI reference methods [3] and also in Roswell Park Medium Institute 1640 medium (RPMI) (Gibco, Thermofischer Scientific, MA, USA). Briefly, a bacterial suspension diluted in MHB or RPMI to give a final organism density of 5.7 log_10_ CFU/mL was added to wells of a microtiter plate containing serial 2-fold dilutions of vancomycin or amikacin. Bacterial growth was recorded after incubation for 18 hours at 35°C.

### Checkerboard assays (CA)

The combined effect of vancomycin and amikacin on the tested *S*. *aureus* strains was first evaluated by CA in MHB and RPMI [4]. Briefly, a bacterial suspension diluted in MHB or RPMI to give a final organism density of 5.7 log_10_ CFU/mL was added to wells of a microtiter plate containing serial 2-fold dilutions of amikacin along the ordinate and serial 2-fold dilutions of vancomycin along the abscissa. After incubation at 35°C for 18 hours, the MIC of both drugs were determined in wells containing one drug and in wells containing the combination of both drugs. The Fractional Inhibitory Concentration (FIC) index was then calculated according to Eq 1.

$$\text{FIC}\;\text{index}=\frac{\text{MIC}\;\text{of}\;\text{amikacin}\;\text{in}\;\text{combination}}{\text{MIC}\;\text{of}\;\text{amikacin}\;\text{alone}}+\frac{\text{MIC}\;\text{of}\;\text{vancomycin}\;\text{in}\;\text{combination}}{\text{MIC}\;\text{of}\;\text{vancomycin}\;\text{alone}}$$ (1)

According to Odds [4], synergism is defined as an FIC index < 0.5, the absence of interaction as an FIC index between 0.5 and 4 and antagonism as an FIC index > 4. These assays were performed in triplicate.

### sTKS (constant antibiotic concentrations)

A few colonies from an overnight culture of *S*. *aureus* were grown at 37°C for 18 h in MHB or RPMI. The bacterial suspension was then centrifuged for 10 min at 3000g and the bacteria were resuspended in fresh pre-warmed MHB or RPMI and diluted to obtain standard (5.0 log_10_ CFU/mL) or large (9.0 log_10_ CFU/mL) bacterial inocula in MHB or RPMI.

Each inoculum size was exposed to no drug (control), 18 μg/mL of vancomycin, 70 μg/mL of amikacin or both for 24 hours corresponding to the maximum free plasma concentrations of the antibiotics when administered at their current dosing regimen. The intravenous administration of 1g of vancomycin is described to lead to a total peak concentration of 32μg/mL [21]. However, since vancomycin binding to plasma proteins is of 45% [22] and since bound vancomycin is not active on bacteria [23], the vancomycin tested concentration in this *in vitro* sTKS experiment was equal to the free maximal plasmatic concentration of 18μg/mL [18]. The administration of 15mg/kg of amikacin is described to achieve a free peak plasma concentration between 60 and 80μg/mL with a negligible binding and we decided to reproduce a peak mean concentration of 70μg/mL [24]. So, in these sTKS experiments, that the highest free drug concentrations achievable in patients were maintained over 24 h in tubes whereas in patients, the concentrations would decrease due to elimination process. The activity of the antibiotics was considered as bactericidal when we observed a reduction of the bacterial burden of more than 3 log_10_ CFU/mL. Samples were collected from each suspension at 0, 2, 4, 8 and 24h to count the viable bacteria. All experiments were performed in duplicate.

### dTKS (fluctuating antibiotic concentrations)

A Hollow-Fiber model was used to assess the antibacterial activity of the combination of amikacin and vancomycin on *S*. *aureus* during exposure to fluctuating clinically-relevant antibiotic concentrations. Basically, the HF model includes a cartridge with capillaries composed of a semipermeable polysulfone membrane. The pore size of the capillaries (42kDa) allows equilibration of the concentrations of chemicals which circulate through the central and peripheral compartments by means of a peristaltic pump (Duet pump, FiberCell Systems, Inc., Frederick, MD, USA) while the bacteria stay confined to the extracapillary space in the peripheral compartment.

Twenty milliliters of a suspension containing 5.0 log_10_ CFU/mL of MSSA were inoculated into the extracapillary space of a hollow-fiber cartridge (C2011 polysulfone cartridge, FiberCell Systems, Inc., Frederick, MD, USA) and incubated at 37°C. The antibiotics were added either 30 min later (standard inoculum assays) or 3 days later (large inoculum assays). For each experiment, the exposure to amikacin and/or vancomycin lasted 5 days to simulate the pharmacokinetic profiles of patients receiving the antibiotics at current dosing regimens i.e. 15mg/kg amikacin once a day [24] and/or 1 g vancomycin twice a day [25] corresponding to maximum free concentrations (C_max_) in the central compartment of 70μg/mL for amikacin and 18μg/mL for vancomycin by considering a plasma protein binding negligible for amikacin and equal to 45% for vancomycin. Previous studies showed that there were no issue with the drug binding to the polysulfone fibers [18,26,27]. The drugs were continuously diluted by means of a second peristaltic pump (MiniRythmic PN+, SMD, Fleury sur Orne, France) to simulate a mean elimination half-life of 4 hours, that could be observed for amikacin and vancomycin in patients [19,20].

One milliliter samples were collected aseptically from the extracapillary space in the HF cartridge to count bacteria 0, 2, 4, 6, 8, 10, 24, 34, 48, 58, 72, 80, 96 and 104h after the first antibiotic administration. The experiments, including controls and exposure to amikacin and vancomycin in monotherapy or in combination, were performed in duplicate for the high inoculum and in one experiment for the standard inoculum. Due to the small number of assays and the small number of bacterial strains used in this study, we could not perform statistical analysis.

The general design of the study is indicated in Table 1.

**Table 1:** General design of the study with all the assays performed on each bacterial strain.

	MSSA		MRSA	
	MHB	RPMI	MHB	RPMI
**MIC**	SI	SI	SI	SI
**Checkerboard assay**	SI	SI	SI	SI
**sTKS**	SI and HI	SI and HI	SI and HI	SI and HI
**dTKS**	NA	SI and HI	NA	NA

SI: Standard Inoculum, HI: High (Large) Inoculum, NA: Not Assessed

### Bacterial counts in TKS

The sampled bacterial suspensions were centrifuged at 3000g for 5 min, the supernatant was discarded, and the pellet was resuspended in NaCl 0.9%. The suspension was then serially diluted and plated in triplicate on tryptic soy agar supplemented with magnesium sulfate and activated charcoal to prevent antibiotic carry-over effects. The colonies were counted after overnight incubation at 37°C. The limit of detection (LOD) was 2.5 log_10_ CFU/mL.

### Statistical analysis

The bacterial counts obtained from 0 to 24 h (sTKS and in dTKS) and from 0 to 104h (dTKS) with the same strain, inoculum size and broth were analyzed with a two-way ANOVA to test the effect of the sampling time and of the treatment (amikacin and vancomycin alone or in combination) with repeated measures (Systat 12 software). When the two-way ANOVA gave a p-value < 0.05 for interaction between the time and treatment factors or for the treatment factor, a pairwise comparison of the different treatments was performed with a Tukey test with a correction for multiplicity.

## Results

### MIC

The MICs of vancomycin and amikacin for the MRSA and the MSSA strains in MHB and in RPMI are given in Table 2. The susceptible or intermediate status of both strains to both drugs, independently to the resistance to methicillin, was targeted to allow the detection of an advantage of the combination.

**Table 2:** MICs (μg/mL) of vancomycin and amikacin for the MRSA and the MSSA strains in MHB and in RPMI.

	MIC of Vancomycin (μg/mL)		MIC of Amikacin (μg/mL)	
	MHB	RPMI	MHB	RPMI
**MRSA ATCC 33591**	1 (S)	1	16 (I)	8
**MSSA HG001**	1 (S)	1	1 (S)	0.5

(S) classified as susceptible according to EUCAST breakpoints

(I) classified as intermediate according to EUCAST breakpoints

Based on the EUCAST breakpoints (standard determination with MHB) [28], both strains were considered as susceptible to vancomycin. The MSSA strain was considered as susceptible to amikacin and the MRSA strain showed an intermediate susceptibility to amikacin.

### Checkerboard assays

To assess the bactericidal activity of a combination of amikacin and vancomycin on *S*. *aureus*, CA were performed in duplicate for the two strains in MHB and RPMI.

The FIC index of amikacin and vancomycin for each of the 2 tested strains, ranged between 1 and 1.5 in both MHB and RPMI, meaning that under the classical conditions of this CA, no interaction (no synergy or antagonism) was demonstrated between these two antibiotics [4].

### sTKS

We then assessed the activity of constant concentrations of vancomycin at 18μg/mL and of amikacin at 70μg/mL, used alone or in combination, for 24hours in MHB and RPMI on a large initial *S*. *aureus* bacterial burden of 8.7 ± 0.4 log_10_ CFU/mL and on a standard inoculum of 4.6 ± 0.4 log_10_ CFU/mL. The stability of both antibiotics in MHB and RPMI over 24h was assessed and no significant degradation of the antibiotics was observed after 24h at 37°C.

**Fig 1:**
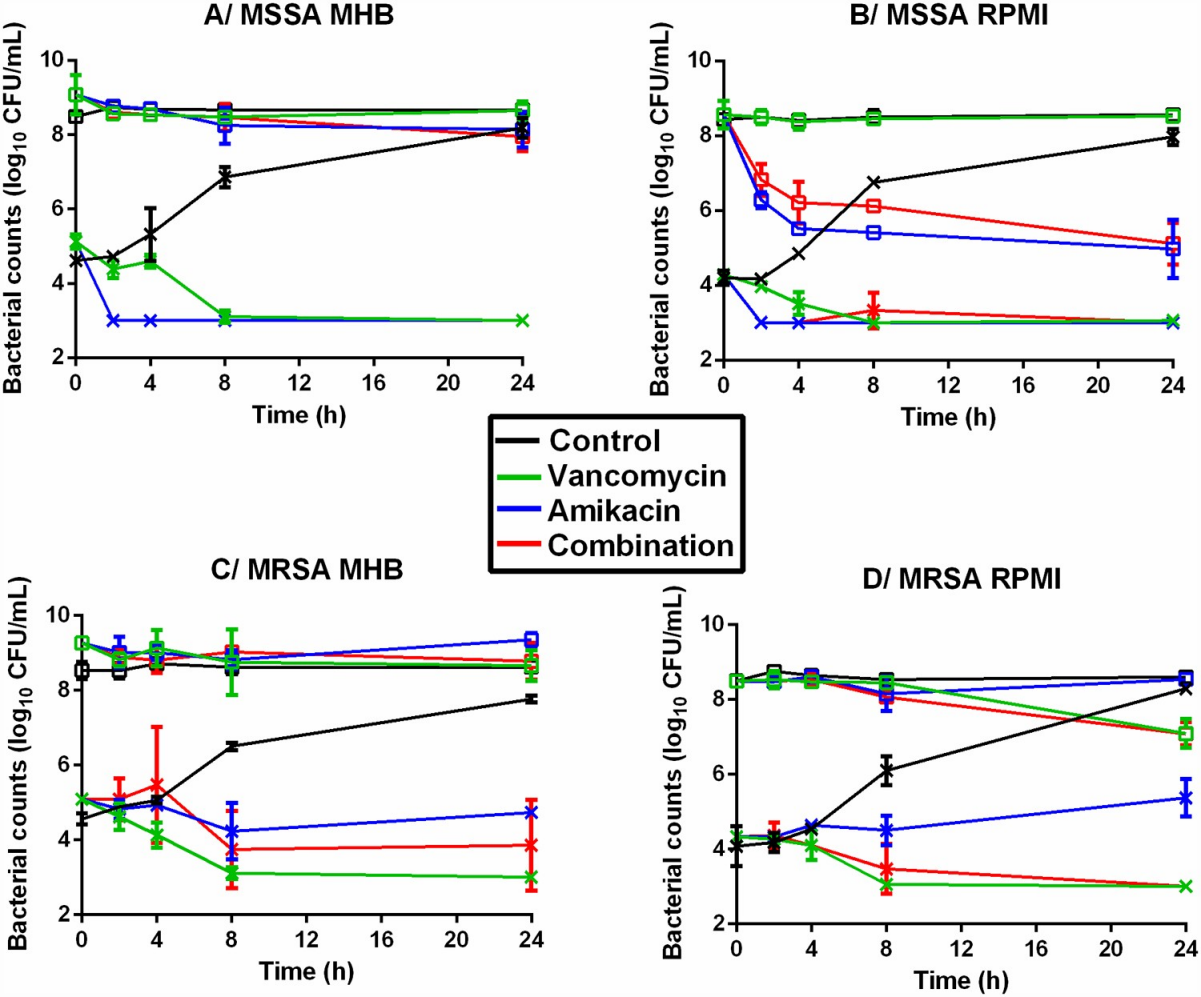
Time-kill curves of the 2 strains subjected to constant concentrations of antibiotic. Evolution of the bacterial population (log_10_ CFU/mL) for the MSSA (A and B) and MRSA (C and D) strains after exposure to 70μg/mL amikacin (blue) or 18μg/mL vancomycin (green) or the combination of both (red) over 24hours in MHB (A and C) and in RPMI (B and D). The marks represent the mean ±SD of the bacterial counts for the different tested treatments (n = 2 for each treatment). Curves with open squares represent the bacterial counts on a large initial inoculum and curves with crosses represent the bacterial counts on a standard initial inoculum. (LOD = 2.5 log_10_ CFU/mL). For the low inoculum of MSSA in MHB and RPMI (A and B), there was no difference between drugs alone or in combination. For the high inoculum of MSSA, there was no difference between drugs in MHB (A) but vancomycin led to significantly higher bacterial counts than amikacin and the combination in RPMI (B). For the low inoculum of MRSA, there was no difference between drugs alone or in combination in MHB (C) but amikacin led to significantly higher bacterial counts than vancomycin and combination in RPMI (D). For the high inoculum of MRSA, there was no difference between drugs in MHB (C) but amikacin led to significantly higher bacterial counts than the combination in RPMI (D).

The time-kill curves obtained by exposing the different inoculum sizes of the two strains to antibiotics are shown in Fig 1.

In absence of antibiotic, the bacterial counts obtained after incubating the initial standard inocula for 24 hours, ranged between 7.8 ± 0.1 log_10_ CFU/mL and 8.3 ± 0.1 log_10_ CFU/mL for both strains in MHB and RPMI. The size of the initial large inoculum remained stable with bacterial counts ranging from 8.6 ± 0.1 log_10_ CFU/mL to 8.8 ± 0.2 log_10_ CFU/mL after 24 hours.

Vancomycin alone was bactericidal on standard inocula of the two strains in MHB and in RPMI since it reduced the bacterial burden to below the LOD after 8 to 24h of antibiotic exposure. Exposure of a large inoculum to vancomycin for 24 h did not reduce the bacterial populations except for the MRSA strain in RPMI where the bacterial population was reduced by 1.4 log_10_ CFU/mL.

Amikacin alone was bactericidal on the standard inoculum of the MSSA strain and reduced the bacterial counts to below the LOD within less than 8h in both media. The reduction of the standard inoculum was smaller for the MRSA strain for which the bacterial counts were 4.8 ± 0.1 and 5.4 ± 0.5 log_10_ CFU/mL in MHB and in RPMI respectively after 24h of exposure. These counts were 3 log_10_ CFU/mL less than the counts in control experiments but close to the initial bacterial counts, thereby demonstrating a bacteriostatic effect. The bacterial populations in large inocula were not reduced by exposure for 24 h to amikacin alone except for the MSSA strain in RPMI where the population was reduced by 3.6 log_10_ CFU/mL.

The combination of amikacin and vancomycin at constant concentrations over 24 hours never showed any significant difference from the better of the two drugs used alone. For the MSSA strain, the combination gave similar results to those obtained with amikacin alone. The bacterial loads after exposure of the standard inocula to the combination for 24 hours were below the LOD. For the large inocula, the bacterial counts were 8.0 ± 0.4 log_10_ CFU/mL and 5.1 ± 0.6 log_10_ CFU/mL after 24h in MHB and RPMI respectively. For the MRSA strain, the combination gave similar results to vancomycin used alone. The bacterial counts after exposure of the standard inocula to the combination for 24 hours were 3.2 ± 0.3 log_10_ CFU/mL in MHB and below the LOD in RPMI. For the large initial inocula, the bacterial counts were 8.8 ± 0.5 log_10_ CFU/mL and 7.1 ± 0.1 log_10_ CFU/mL after 24 hours in MHB and RPMI respectively.

### dTKS

The HF model was applied to assess the efficacy of vancomycin and amikacin used alone or in combination under dynamic conditions over 5 days in RPMI on a standard inoculum of 5.4 ± 0.3 log_10_ CFU/mL and on a large inoculum of 9.4 ± 0.3 log_10_ CFU/mL.

In dTKS, the antibiotic concentrations fluctuated over time so as to resemble the concentration profiles observed in patients receiving repeated recommended doses of amikacin and vancomycin over 5 days. Vancomycin was administered twice a day at a C_max_ of 18μg/mL and amikacin was administered once a day at a C_max_ of 70μg/mL. Both antibiotics were continuously diluted to simulate an elimination half-life of 4 hours.

In the absence of antibiotic (control experiments), the standard inocula increased to 9.5 ± 0.4 log_10_ CFU/mL in 24h. Thereafter, all the inocula (standard and large) increased slightly over the next 4 days to reach a final bacterial count of 10.8 ± 0.2 log_10_ CFU/mL at the end of 5 days.

**Fig 2:**
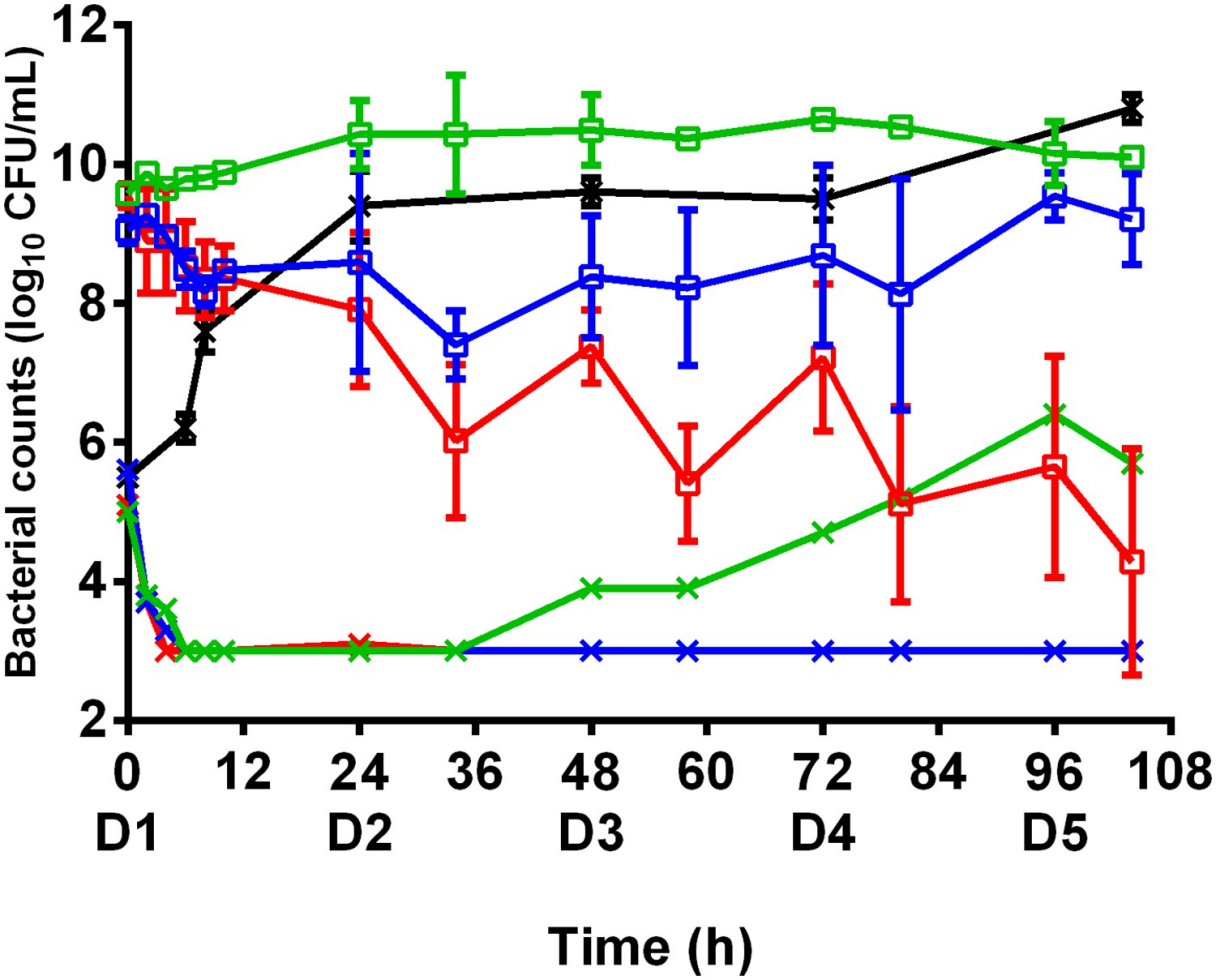
Time-kill curves of the MSSA strain subjected to dynamic concentrations of antibiotic. Evolution of the bacterial population (log_10_ CFU/mL) after exposure to amikacin or vancomycin alone or the combination of both over 5 days in RPMI. The marks represent the mean ±SD of the bacterial counts for the different tested treatments (black: control without antibiotic, green: vancomycin twice a day, blue: amikacin once a day, red: amikacin once a day and vancomycin twice a day. Curves with open squares represent the bacterial counts on a large initial inoculum and curves with crosses represent the bacterial counts on a standard initial inoculum. (n = 2 for each treatment on the large inocula and n = 1 for each treatment on the standard inocula) and LOD = 2.5 log_10_ CFU/mL). The bacterial counts from 0 to 24 h after exposure to vancomycin were significantly higher (p<0.01) than those obtained with amikacin or the combination. The bacterial counts from 0 to 104 h were significantly different for each treatment (p<0.01).

The time-kill curves for bacteria exposed in the HF model to amikacin and vancomycin alone or in combination are shown in Fig 2.

On standard inocula, both antibiotics, alone or in combination, decreased the bacterial burden to below the LOD within less than 8h, as had been obtained with a constant antibiotic concentration in sTKS. When exposed to amikacin alone or in combination, the bacterial population remained below the LOD until the end of the experiment whereas the bacterial population increased from 34h onwards until it was 6.4 log_10_ CFU/mL at the end of the experiment with vancomycin alone.

Large inocula of bacteria exposed to fluctuating concentrations of vancomycin alone were never reduced, as in sTKS. After exposure for 5 days, the population size in the HF model was comparable to that of control experiments, the final bacterial count being 10.0 ± 0.1 log_10_ CFU/mL. Exposure to fluctuating concentrations of amikacin alone led to a slight reduction of the bacterial population over the first 24 hours, as had been observed with constant amikacin concentrations in sTKS. However, the reduction of 0.9 log_10_ CFU/mL observed on the first day in the HF model was quite slight and, after 5 days of treatment, the final bacterial count of 9.2 ± 0.7 log_10_ CFU/mL was very similar to that of the initial population and not much lower than in control experiments.

When amikacin and vancomycin were used in combination in the HF model, a decrease of the bacterial population was observed over the first 24 hours, similar to the treatment with amikacin alone, with bacterial counts of around 8 log_10_ CFU/mL. However, significant different results (from amikacin alone) were obtained after exposure to the combination for 5 days, the mean final bacterial count of 4.3 ± 1.6 log_10_ CFU/mL corresponding to an overall reduction of more than 5 log_10_ CFU/mL.

This observation of bactericidal activity with the combination after 5 days and no or only slight activity with both drugs used alone demonstrated a synergy between amikacin and vancomycin for *S*. *aureus* in the HF model.

## Discussion

In this study, our objective was to compare the results of antibacterial efficacy of drug combination obtained by performing checkerboard assays (CA), static Time-Kill studies (sTKS) or dynamic Time-Kill Studies (dTKS). For this comparison, we selected two antibiotics used for the management of *S*. *aureus* infection, namely amikacin and vancomycin. The activities of the two drugs, alone or in combination, were investigated on two different inoculum sizes of two *S*. *aureus* strains susceptible to vancomycin and susceptible or intermediate to amikacin. The efficacy of the antibiotic combination on the tested *S*. *aureus* strains was found to differ depending on the inoculum size and the methods used.

The susceptibility of both strains to vancomycin was confirmed in sTKS by the systematic eradication of a standard inocula exposed to the concentrations of vancomycin achievable in patients, and in dTKS by a decrease of bacterial counts of the standard inocula below the LOD after 8 to 34 hours. In these dynamic experiments, slight bacterial regrowth was observed which attained 7 log_10_ CFU/mL after 5 days, despite repeated administrations of vancomycin twice a day. However, because no bacteria could be detected during 24 hours, we hypothesized that, *in vivo*, the immune system in immunocompetent patients could take over from antibiotic action to eradicate the bacteria, prevent regrowth and lead to a bacterial cure. Testing two inoculum sizes revealed an important effect of inoculum size for vancomycin, since no bactericidal activity (i.e. a reduction of more than 3 log_10)_ was demonstrated on large bacterial inocula of the two susceptible strains in either culture medium. One hypothesis for this effect is that bacteria are able to trap and rapidly decrease free vancomycin concentrations in the medium. Cui *et al*. [29] described this phenomenon on MRSA and suggested that the thickened peptidoglycan layers of MRSA could trap free vancomycin. Yanagisawa *et al*. [30] also demonstrated that when 4 μg/mL of vancomycin was added to a 7 log_10_ CFU/mL culture of a MRSA strain, the concentration of free vancomycin immediately fell from 4 to 3.5 μg/mL suggesting that the initial trapping by bacteria occurred very rapidly. Other *in vitro* studies also confirmed this inoculum effect on vancomycin susceptible MSSA [31] and on vancomycin highly resistant MRSA strains [32].

For amikacin, the MSSA strain was classified as susceptible whereas the MRSA strain showed an intermediate susceptibility according to the EUCAST MIC breakpoint [28]. For aminoglycosides, the *f*C_max_/MIC ratio (peak free plasma concentration divided by the MIC) is considered as the best index to predict the efficacy of a treatment [33] and targeting a breakpoint value of more than 8 to 10 for this ratio is usually recommended to ensure clinical efficacy against the pathogen [34]. When the amikacin concentration tested in sTKS was 70 μg/mL, the ratios of the tested concentration over the MIC were 70 and 140 in MHB and RPMI respectively for the MSSA strain, i.e. far higher than the breakpoint value for clinical efficacy. Indeed, the standard bacterial inoculum was rapidly eradicated after exposure to amikacin in both sTKS and dTKS. For the MRSA strain, the fC_max_/MIC ratios were 4.4 and 8.8 over the 24h of exposure in MHB and RPMI respectively and were lower than or very close to the breakpoint value, suggesting that antibacterial activity would be uncertain. Our results confirmed that amikacin exhibited bacteriostatic but not bactericidal activity on a standard inoculum of the MRSA strain in the two media.

Large inocula of MSSA and MRSA bacteria were not eradicated by amikacin in the sTKS. Thus, the classification of these bacteria as susceptible or intermediate to amikacin, based on the sole MIC determination, could not be predictive of an antibacterial effect on a large inoculum in sTKS. Similar results had been obtained in previous studies when a large inoculum of susceptible *S*. *aureus* was challenged by another aminoglycoside [35,36].

Our results confirmed that no eradication of the bacterial burden was attained with monotherapy in either medium when the inoculum was large.

To assess the potential interest of combining drugs, we first performed a CA which is a derivative of the MIC determination for drug combinations. This assay, classically carried out with a standard inoculum, indicated here indifference between amikacin and vancomycin. This corroborated the sTKS and dTKS results, indicating that the combination did not exhibit better antibacterial activity against standard inocula than the better drug used alone. For large inocula, the assessment of combination efficacy cannot be performed by a standardized CA since this method, like the MIC determination, relies on an assessment of turbidity after incubation for 16–20 h and the initial inoculum therefore needs to be clear and generally below 7 log_10_ CFU/mL. This means that the efficacy of a combination or of one drug alone can only be determined by TKS when the inoculum is large.

With standard inocula, amikacin and vancomycin alone or in combination decreased the bacterial burden to below the limit of detection in less than 24h in sTKS, except for amikacin on the MRSA strain. Reductions of large bacterial inocula were only observed after exposure of the MRSA strain to vancomycin alone or in combination and after exposure of the MSSA strain to amikacin alone or in combination. Thus, in sTKS, the combination never showed a significant different activity from the better drug used alone and never improved the efficacy or rate of bacterial killing over monotherapy, as already shown by the CA results.

Classical sTKS have limitations to mimic *in vivo* conditions due to the brief period of bacterial exposure to the drugs (usually less than 24 h due to the absence of broth renewal) and the constant concentrations of antibiotic over time. The use of constant concentrations, especially of antibiotics with short half-lives, may overestimate drug efficacy since the bacteria are continuously and artificially exposed to the peak concentration. sTKS could also be performed with lower antibiotic concentrations encountered *in vivo* at different times after the peak concentration. However, these experiments would probably not be more representative of the *in vivo* situation since, *in vivo*, the bacteria exposed to lower concentrations have already been confronted to the peak drug concentrations with a possible development of phenotypic or genotypic adaptations contrarily to the conditions of TKS in which the bacteria would be naïve (never confronted to drug before). The principal interest of the dynamic model is to focus on the same initial inoculum over time, with the same bacteria exposed to fluctuations of concentrations over time, as it occurs *in vivo*. Another key-point is the length of the experiment which can be a crucial issue if the aim is to reveal a slow bactericidal effect, tolerance or amplification of resistance. In this study, the dTKS with the HF model were conducted over 5 days, but weeks or months have also been described in other experiments using the same model [27,37]. With the large inoculum, similar results were obtained for sTKS and dTKS over the first day i.e. bactericidal activity of amikacin and no significant difference between the combination and amikacin alone, which also agreed with the results obtained with the CA. However, some effects on the large inoculum were observed from the second to the fifth day in dTKS, that were not expected from the observation of the CA or sTKS results. The activity of amikacin alone decreased over the days and the final bacterial load was the same as in the initial inoculum. Reduced amikacin activity can be due to tolerance, also known as adaptive resistance [38,39], or to the selection of resistance by repeated exposure to fluctuating concentrations over 5 days [26] but we did not explore further these hypotheses and were unable to discriminate between them.

In contrast, from the second to the fifth day, the vancomycin and amikacin combination produced a progressive decline of the bacterial population and a final reduction of 5.1 log_10_ CFU/mL, compared to the initial bacterial load. This late enhancement of the bactericidal activity might result from a sequential activity of amikacin and vancomycin, the activity of amikacin on the first day reducing the bacterial population and thereby improving the subsequent efficacy of vancomycin. So, performing dTKS with fluctuations of antibiotic concentrations over several days might be helpful to understand therapeutic failures even though a limitation of dynamic *in vitro* models is the absence of consideration of the immune system. However, by using a eukaryotic cell culture medium in sTKS experiments it should be possible to add components of the immune system in further studies.

## Conclusion

The use of HFIM to assess the efficacy of antibiotic combinations is quite poorly represented in the literature and to our knowledge, this study is one the first comparing sTKS and dTKS with two inoculum sizes in different culture media. By exploring the combination of amikacin and vancomycin on two *S*. *aureus* strains, we showed that for low bacterial loads, all the tested methods including rapid methods such as MIC determination and CA led to similar results. However, for high bacterial loads, longer experiments with fluctuating concentrations of drugs revealed antibacterial effects varying over days which were not observed with shorter experiments. These results suggested that the efficacy of drug combination on infections associated with a high bacterial load could be difficult to assess and that the relevance of dynamic models to predict *in vivo* efficacy should be investigated to further promote the test of combinations in such models.
